# Improved Identification of Small Open Reading Frames Encoded Peptides by Top-Down Proteomic Approaches and De Novo Sequencing

**DOI:** 10.3390/ijms22115476

**Published:** 2021-05-22

**Authors:** Bing Wang, Zhiwei Wang, Ni Pan, Jiangmei Huang, Cuihong Wan

**Affiliations:** Hubei Key Lab of Genetic Regulation and Integrative Biology, School of Life Sciences, Central China Normal University, No. 152 Luoyu Road, Wuhan 430079, China; wangb@mail.ccnu.edu.cn (B.W.); wzw123456@mails.ccnu.edu.cn (Z.W.); panni407666377@mails.ccnu.edu.cn (N.P.); jm_huang@mails.ccnu.edu.cn (J.H.)

**Keywords:** sORF-encoded peptides, de novo sequencing, top-down, non-ATG start codon, sequence coverage

## Abstract

Small open reading frames (sORFs) have translational potential to produce peptides that play essential roles in various biological processes. Nevertheless, many sORF-encoded peptides (SEPs) are still on the prediction level. Here, we construct a strategy to analyze SEPs by combining top-down and de novo sequencing to improve SEP identification and sequence coverage. With de novo sequencing, we identified 1682 peptides mapping to 2544 human sORFs, which were all first characterized in this work. Two-thirds of these new sORFs have reading frame shifts and use a non-ATG start codon. The top-down approach identified 241 human SEPs, with high sequence coverage. The average length of the peptides from the bottom-up database search was 19 amino acids (AA); from de novo sequencing, it was 9 AA; and from the top-down approach, it was 25 AA. The longer peptide positively boosts the sequence coverage, more efficiently distinguishing SEPs from the known gene coding sequence. Top-down has the advantage of identifying peptides with sequential K/R or high K/R content, which is unfavorable in the bottom-up approach. Our method can explore new coding sORFs and obtain highly accurate sequences of their SEPs, which can also benefit future function research.

## 1. Introduction

Small open reading frames (sORFs) and their encoded peptides (SEPs) with less than 100 amino acids are overlooked due to the arbitrary definition for protein-coding regions in conventional genome annotation pipelines [1,2]. Of the human genome, 98% is considered to be transcribed into noncoding RNAs (ncRNAs) [3]. However, the coding potential of these noncoding genes has been re-evaluated, especially for sORFs [4,5,6]. Increasing evidence demonstrates that these SEPs play critical roles in various biological processes, including the metabolism, translation regulation, development, and biomarkers of pathologies [7,8,9,10]. Thanks to the rapid development of RNA sequencing techniques [11,12], mass spectrometry (MS)-based proteogenomics, and bioinformatics [13,14], SEP discovery has changed from the occasional case study to genome-wide research [15], which resulted in the breakthrough of functional SEP analysis.

Most current MS-based proteogenomics in SEP identification use a bottom-up strategy to obtain peptides with moderate length in the range of 10 to 20 AAs [16,17]. However, many SEPs have high sequence overlap with annotated coding sequence regions. Therefore, it is hard to find unique or specific peptides for these SEPs by a bottom-up approach. It is worth noting that some reported SEPs have suitable lengths [18] for direct peptidomics determination. Current SEP identification tends to find relatively larger peptides (50–100 AAs), while shorter peptides (≤50 AAs) dominate the predicted database [4,19]. These deficiencies in the bottom-up strategy may interfere with the accurate identification of novel SEPs, thus stimulating our interest in the top-down analysis of these small proteins. Top-down proteomics is arguably the most powerful method for analyzing proteoforms, including those arising from post-translational modifications (PTMs), genetic variations including polymorphisms and mutations, and the alternative splicing of RNA transcripts [3,20].

On the other hand, suitable databases containing all the predicted sequences of SEPs benefit their identification. However, database searches miss many peptides that are not present in the chosen database. Peptides may contain mutations and represent novel protein-coding loci and alternative splice forms. In turn, MS data processed by de novo sequencing can be used to provide protein-level evidence of gene expression and refine gene models [21]. In this work, we present large-scale SEP identification in the Hep3B cell line by integrating top-down and de novo approaches. Novel SEPs were identified, and some of them had complete full-length sequence coverage. The discovery of this hidden proteome may shed light on the complicated regulation mechanism in various biological processes.

## 2. Results

### 2.1. Workflow Overview for SEP Identification

**Figure 1:**
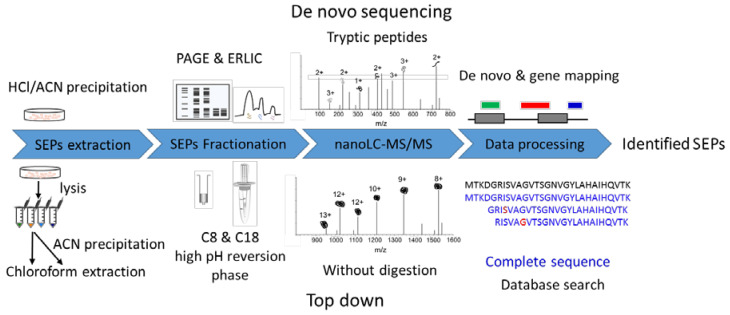
Workflow for SEP identification. De novo sequencing strategy: raw data from bottom-up experiment loaded to pNovo for sequencing, then the de novo specific peptides were filtered and mapped to the genome to define new open reading frames, obtaining entire sequences of SEPs. Top-down strategy: SEPs were enriched, fractionated, and analyzed by liquid chromatography-tandem mass spectrometry (LC/MS/MS) without further digestion. Raw data were searched against a constructed database including proteins in UniProt and OpenProt database to identify SEPs.

Consequently, we propose an optimized approach comprising de novo peptide sequencing and a top-down strategy to analyze SEPs (Figure 1). sORFs are distributed over a wide variety of transcripts and classified into different types according to their genomic locations (Figure S1). Some predicted peptides from ncRNA have identical or highly homologous sequences with the annotated proteins. Therefore, a full-length or high coverage sequence identification of these SEPs provides robust evidence for their existence. The top-down approach is used for native protein identification and full-length sequencing, providing more efficient sequence information for SEPs. Meanwhile, some SEPs are still unknown and not recorded in any databases. Database-independent searches may correct this omission and provide a more comprehensive view of the proteome.

The de novo approach used our previous MS raw data generated by the bottom-up strategy [22]. Most de novo experiments are the same as those for the bottom-up approach, only replacing the database search with de novo sequencing and adding a gene mapping step after that (Figure 1). The top-down raw data were first obtained in this work. Researchers have developed multiple sample preparation methods for bottom-up SEP identification [16,19], but few attempts have been made in top-down SEP identification. To obtain high-quality top-down datasets, we first optimized sample preparation. Biological matrices in vivo, especially lipids, may cause fouling problems in peptide enrichment and separation platforms. Therefore, we performed chloroform extraction resulting in an insoluble substance sharply located at the interface of the two phases. The following MS raw data also demonstrated that our method could effectively extract peptides with a mass range from 1000 to 12,000, just covering the SEP (≤100 AAs) mass range (Figure S2).

Multidimensional separation combined with MS analysis can significantly promote depth of proteome. Here, we compare different enrichment and fractionation methods in improving SEP identification (Figure S3). Low pH reversed-phase C8 and C18 column pre-fractionation can improve detection sensitivity by reducing the sample complexity compared to single-shot analysis. However, high and low pH reversed-phase pre-fractionation has a good orthogonal fractionation effect without repeated desalination [23]. The heat map of identified peptides in different fractions demonstrated the provided orthogonal separation by a high pH reversed-phase micro-column, resulting in boosting SEP identification. Different sample preparation methods were complementary to improved top-down SEP identification, while acetonitrile (ACN) precipitation combined with high pH reversed-phase pre-fractionation was superior to other methods.

### 2.2. Confident SEPs Identified by Top-Down Analysis

In total, 740 peptides corresponding to 241 SEPs were identified by a top-down strategy, and 16 of these SEPs had complete full-length sequence identification (Table S1). As mentioned above, the predicted SEPs have high sequence similarity with other proteins. Thus, full-length SEP detection may provide robust evidence for the expression of these SEPs.

**Figure 2:**
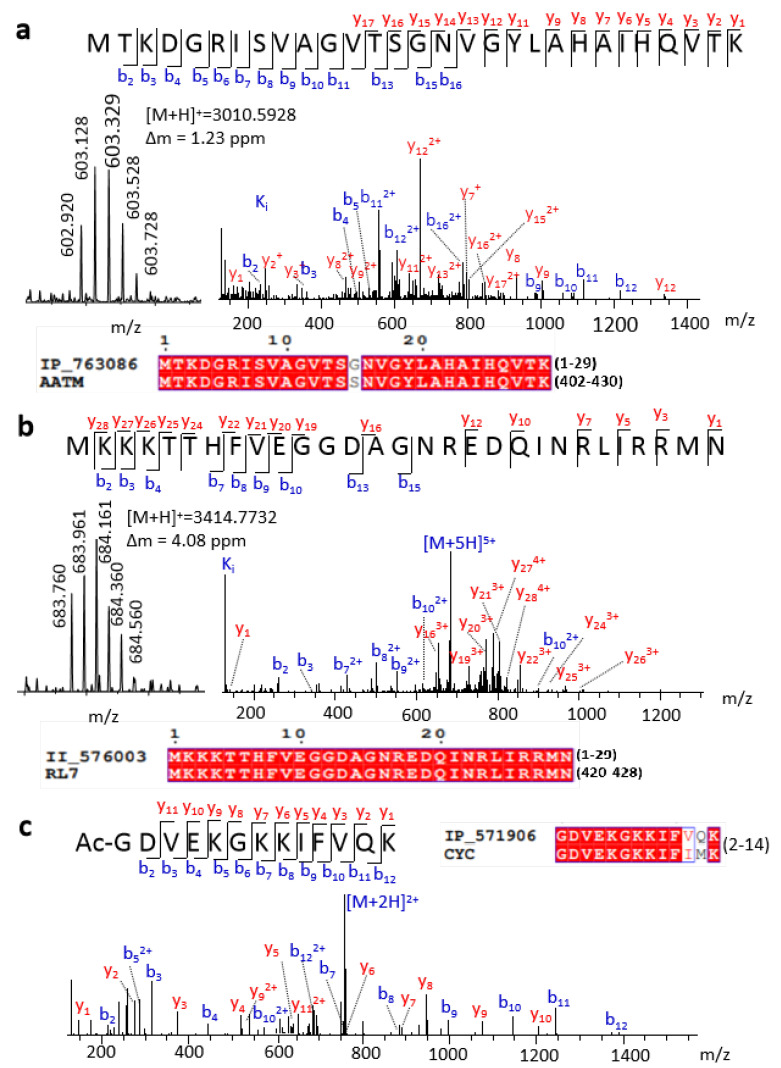
Representative SEPs identified by a top-down approach. Spectra and sequence alignment of (**a**) full length IP_763086, (**b**) full length II_576003, and (**c**) a peptide from IP_571906.

In this work, we detected 16 full-length SEPs. For example, IP_763086 had high sequence similarity to the C-terminal of aspartate aminotransferase, with only one different amino acid (Figure 2a). According to previous gene annotation, the ORF of this peptide is located at chromosome 12 (9,642,922–9,643,011), which belongs to ncRNA, without MS or translation evidence. Our experimental MS precursor ion and abundant tandem mass spectrometry (MS/MS) fragment sequence ions both provided strong evidence for the expression of this SEP (Figure 2a). We also detected the full length of the known protein’s isoform, for example, II_576003 with ORF located at chromosome 8 (73,291,044–73,291,133). II_576003 has an identical sequence with the C-terminal region of longer protein ribosomal protein L7. High-quality MS spectra demonstrated the existence of this peptide sequence (Figure 2b). Truncated forms of known proteins also play crucial biological regulation roles. For example, the truncated variants of EEF1A1 promote or suppress cancer cells [24]. It should be highlighted that this kind of SEP can be confirmed by a top-down rather than a bottom-up approach.

Besides full-length detection, the top-down method has an advantage in detecting PTMs of peptides. The removal of the initiator methionine and N-terminal acetylation is a highly conserved and widespread phenomenon for most proteins that mainly occurs co-translationally [25]. For example, Ac-GDVEKGKKIFVQK, an N-terminal peptide of IP_571906, was first detected (Figure 2c). This peptide has four Lys, which were hard to detect if we treated it with trypsin digestion. Conversely, abundant K or R can improve the proton affinity of the peptides, resulting in enhancement of ionization efficiency and detection sensitivity in MS for this type of SEP. Therefore, a top-down approach will be a benefit for the identification of SEPs of this kind. Except for N-terminal acetylation, some SEP fragments were detected with mass shift when using TopPIC searching (Figure S4), which means potential unknown modifications or partial amino acid mutation.

**Figure 3:**
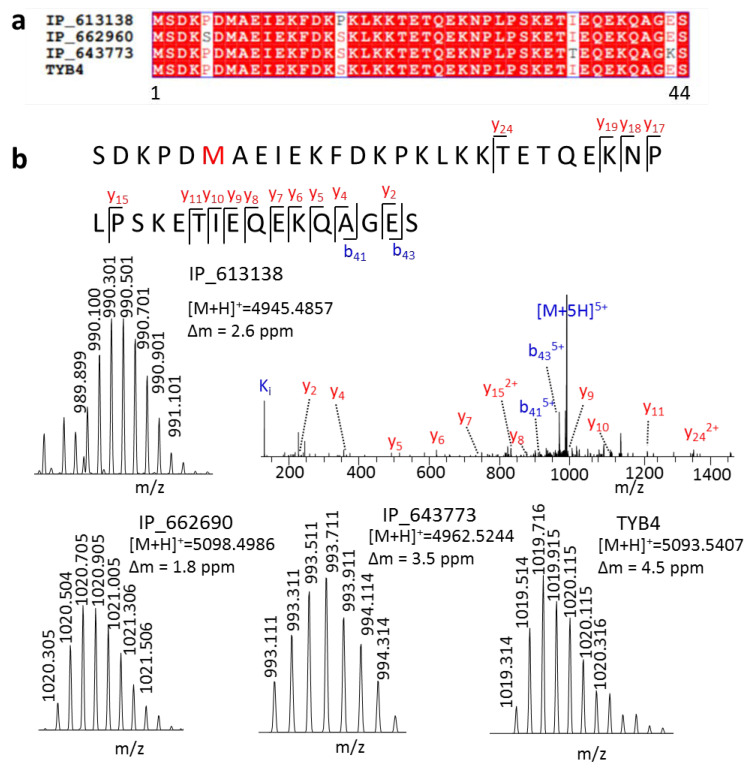
Distinct highly similar peptides by a top-down approach. (**a**) Sequence alignment of peptides IP_613138, IP_662690, IP_643773, and TYB4, and (**b**) their spectra.

Furthermore, we detected three isoforms of TYB4 that had only one or two amino acid differences from TYB4 (Figure 3a). These peptides have many Lys or Arg in their amino acid composition, making it hard to generate ideal tryptic peptides in a bottom-up strategy. Therefore, no MS evidence of these SEPs was found in the OpenProt [4] database. We provided the first MS evidence for these peptides. At first, even with only one amino acid variation, they generated parent ions with different m/z values, which could be easily distinguished in MS1 (Figure 3b). Then, with the fragment ion in MS2, we could also obtain their full sequence.

### 2.3. Boost Sequence Coverage by a Top-Down Approach

**Figure 4:**
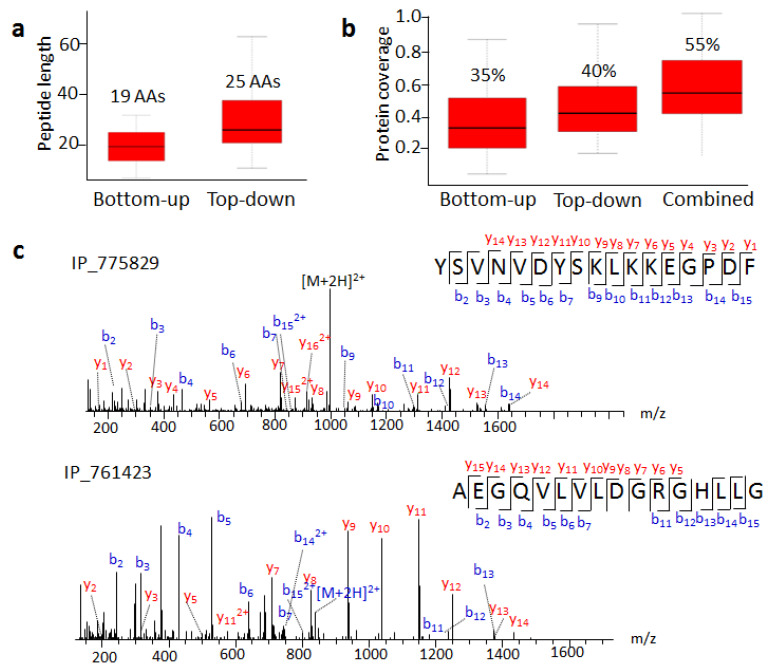
Top-down approach improves SEP identification. (**a**) Average lengths of peptides identified by bottom-up and top-down approaches. (**b**) SEP sequence coverage of bottom-up and top-down approaches. (**c**) MS/MS spectra of IP_775829 C-terminal and IP_761423 N-terminal peptides.

Seventy SEPs we detected by the top-down approach have MS evidence according to the OpenProt database. We compared the length of detected peptides and coverage for these SEPs in these two strategies (Table S2). The average peptide length with the bottom-up strategy was 19 AA, while that with the top-down approach was 25 AA (Figure 4a). The longest peptide from the bottom-up or top-down approach had 30 or 76 AA, respectively. Combined bottom-up results from different datasets were able to provide 35% sequence coverage without full sequence coverage. On the other hand, the top-down approach in our work had an average of 40% sequence coverage (Figure 4b), and the highest peptide coverage was 100%. Undoubtedly, the top-down approach increased the length of retrieved peptides significantly more than the bottom-up approach, thus improving sequence coverage and identification confidence.

Furthermore, integrating the bottom-up and top-down results vastly improved sequence coverage (Figure 4b). For example, IP_759730 is a 92 AA SEP with only four different amino acids from HMGA1. We detected its N-terminal peptide containing one differential amino acid. With previous MS evidence and our result, its sequence coverage increased from 19.6% to 47.8% (Table S2). The detection of more unique peptides containing differential sites indicated higher confidence for SEP expression.

The SEPs degraded into small pieces, which resulted in sequence coverage that was not always 100% in the top-down approach. We detected peptides belonging to the N- or C-terminal region of the predicted SEPs. For example, we detected the C-terminal fragment of IP_775829, an 82 amino acid long SEP, containing 17 amino acids (Figure 4c). IP_761423 had MS evidence with a tryptic peptide AEGQVLVLDGR in the OpenProt database. Here, we detected a longer N-terminal peptide, AEGQVLVLDGRGHLLG (Figure 4c). After analyzing C- and N-terminal regions flanking peptides split by precursor SEPs, we found that the most abundant residues trailing the C-termini were Lys, while Ser and Gly occupied the dominant proportion at N-termini (Figure S5). This agreed with the consensus that Gly or Ser residues are involved in the cleavage mechanism of many different kinds of proteolytic approaches [26].

### 2.4. Novel SEPs Identified by de Novo Sequencing

**Figure 5:**
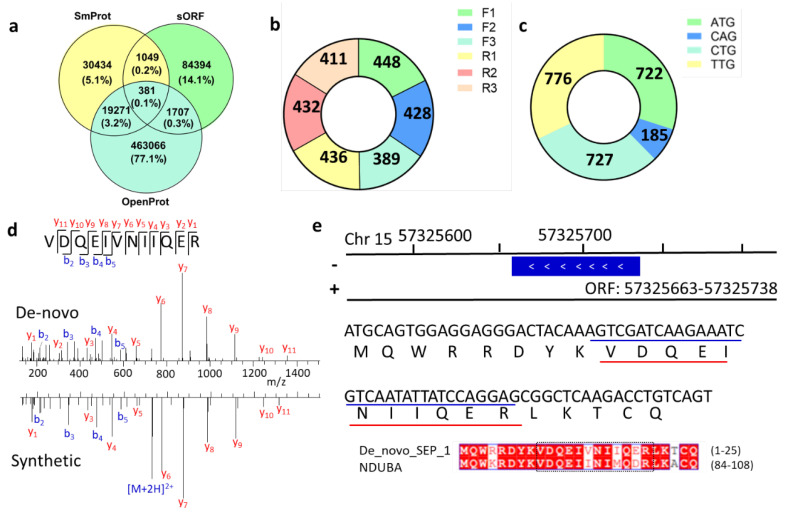
Novel SEPs identified from de novo sequencing. (**a**) Overlap among different SEP databases. (**b**) Distribution of reading frame shift of SEPs. (**c**) Numbers of novel SEPs with different start codons. (**d**) MS/MS spectra of VDQEIVNIIQER peptide. (**e**) Genome location and full sequence of de novo sequencing predicted peptide in (**d**).

By comparing the predicted SEP sequences from different databases (OpenProt [4], SmProt [5], sORFs [6]), we found low overlap among them (Figure 5a). The uncertainty of this ghost proteome stimulated our interest in a novel implementation of peptide de novo sequencing in combination with proteogenomics tools to refine genome annotation. MS/MS spectra that were ignored in database searches but survived in de novo sequencing were further considered. High-confidence de novo peptides with high peptide-spectrum match (PSM) scores were selected and further filtered by BLASTp to delete peptides with identical amino acid sequences to reference proteins. The 1682 remaining peptides were mapped to 2544 sORFs (Table S3) that were randomly located on all chromosomes (Figure S6). Some peptides matched multiple ORFs with different full lengths. Like traditional proteomics, one cannot tell the difference among protein groups without unique peptides. So, further experiments are needed to confirm which one is the actually expressed gene.

The Proteogenomic Mapping Tool [27] matches these unique peptides against databases generated from the genome translated in all six reading frames. The ribosome can shift the reading frame by attaching at +2 or +3 of the start codon, thus allowing for the translation of AltProt. By calculating the SEPs generated from reading frame shift, we found that the frameshift mutation may be a non-negligible source of SEPs (Figure 5b). About half of these novel SEPs were exclusively allocated to non-annotated antisense transcripts. Currently, OpenProt does not consider ORFs with non-ATG start codons. However, translation can be initiated at near-cognate start codons rather than the classical AUG [28], and our results had similar observations as previous research. After evaluating the start codons of these novel SEPs, it was found that more than two-thirds of their ORFs are initiated with non-ATG start codons (Figure 5c).

To validate the de novo sequencing results, we randomly chose several peptide sequences and synthesized them. Mass spectra from de novo sequencing and synthesized peptides were highly consistent (Figure S7), suggesting that the results of de novo sequencing were accurate. For example, the match of the experimental MS spectrum to synthetic peptide spectrum demonstrated the existence of a novel unique peptide, VDQEIVNIIQER (Figure 5d). After gene mapping, we found that the corresponding ORF of this peptide was allocated at Chr 15: 57,325,663–57,325,738 (Figure 5e). Its coding SEP full sequence is MQWRRDYKVDQEINIIQERLKTCQ, which was highly similar to the sequence of protein NDUBA by BLASTp.

### 2.5. Characteristics of Total Identified SEPs

Finally, combining top-down and de novo sequencing, we obtained 2422 peptides that belonged to 2785 SEPs in total. With our results, we confirmed 171 predicted SEPs by the top-down approach and provided evidence of 1682 peptides from novel SEPs by de novo sequencing. All SEPs found by de novo sequencing were from ORFs in the noncoding ranges and did not have annotations or other information in the SEP databases. So, we mainly analyzed the characteristics of SEPs from the top-down and database-dependent search according to OpenProt [4] annotation (www.openprot.org, version number 1.5, 10 August 2020).

Some full-length detected SEPs were less than 50 amino acids, while most of these newly detected SEPs were longer than 50 amino acids (Table S1, Figure S8). Almost 70% of the top-down SEPs with ORFs originated from ncRNA (Figure S8). About one-third of the top-down SEPs had MS or translation evidence (Figure S9), and our data further supported the possibility of their existence. Several SEPs corresponded to sORFs that had Kozak motif or a high-efficient translation initiation site (Table S1), which suggested high translation efficiency. Our data also showed that SEPs from sORFs containing these motifs tend to have higher PSMs.

Over 80% of those SEPs contain predicted domains (Figure S9). Domain is a functional unit of protein. Being rich in domains indicated that these SEPs might have related functions. More than half of SEPs have high conservation across different species (Figure S9). Evidence of evolutionary conservation is essential because sORFs that lack cross-species conservation are more likely to be random sequences that do not encode functional peptides [29,30]. In turn, most recently discovered functional SEPs have high conservation among different species [31,32]. Furthermore, some of these SEPs have high homology to the reference proteins and may play similar or competitive roles in various biological processes.

## 3. Discussion

In this study, we demonstrated an improvement in SEP identification by using top-down and de novo sequencing. Several endogenous full-length SEPs were detected, which is consistent with previous ribosome sequencing results, shedding light on the existence of this hidden proteome.

We detected many degradative fragments from proteins besides SEPs. Proteolysis is ubiquitous in vivo all the time, resulting in SEPs being “buried” by the vast number of peptides generated by the degradation of known proteins. Note that the N-terminal or C-terminal peptides tended to frequently appear; that is also an important indicator for the initiation or termination of the translation process. We detected widespread N-terminal acetylation on these SEPs, which is a very common phenomenon for the initiation stage of translation [33]. However, the detected unique peptides improved the sequence coverage for SEPs, thus enhancing identification confidence. On the other hand, small proteins tend to lack a stable higher structure, and the loose structure is more likely to be recognized by the widespread hydrolase. The determination of large amounts of protein fragments instead of full-length SEPs indicates a short half-life or rapid degradation for this kind of small protein. It may suggest that the low abundance of SEPs in the cells was due both to low expression and rapid degradation.

For a few SEPs, the intensity of fragment sequence ions was low. The low fragmentation efficiency is caused by the lack of mobile proton and salt bridge. The collisional activation process originates from collisions between ions and inert gas molecules, resulting in converting kinetic energy into internal energy and triggering fragmentation [34]. Due to the limited energy input, this popular method has distinct limitations in large peptides or proteins. Combining other fragmentation technologies such as electron capture dissociation (ECD) and electron transfer dissociation (ETD) will further benefit the top-down analysis of SEPs [34].

The implementation of peptide de novo sequencing in combination with a proteogenomics tool provided novel predicted sORFs. Long peptides will provide high confidence for novel sORFs prediction. However, most filtered de novo specific peptides are short due to the tendency of current de novo sequencing software to be highly accurate for short peptides [35]. The average length of de novo peptides is 9 AA (Table S2). However, this approach can also provide valuable information for finding novel sORFs. Using different enzymes in the digestion process and integrating the peptides with overlapping sequences generates long unique peptides. That may significantly improve the accurate prediction of novel sORFs. The other problem is that many de novo peptides cannot match any ORF with current algorisms. This may be caused by a rare initiation codon, mutation, or splicing. Alternatively, improved gene mapping algorisms are needed to dock with de novo sequencing.

It is widely accepted that sORFs and SEPs could represent steps in gene, peptide, and protein evolution [30]. Functional and species conservation analyses suggest that these AltProts may provide an abundant resource for the birth of new functional proteins. The identification of homologs of a candidate protein can provide strong evidence of its function. As shown in this work, most of the novel identified SEPs were homologous to known proteins. Meanwhile, the conservation of ORFs across different species can also help to identify functional SEPs that have been preserved by natural selection [36]. Nevertheless, it is interesting to note that the functions of reported known SEPs rely on interactions with larger known annotated proteins. Peptide motifs mediate a large portion of protein-protein interactions in different cellular processes. Overall, identifying confident novel SEPs with high sequence coverage is significant for illustrating the generation mechanism for AltProts, and is fundamental for further functional analysis.

## 4. Materials and Methods

### 4.1. Cell Culture

Hep3B cells were cultured in a DMEM medium with 10% fetal bovine serum (FBS), penicillin, and streptomycin on a 10 cm dish (Corning Inc., Corning, NY, USA). All cells were grown at 37 °C under 5% CO_2_ using the HF90 carbon dioxide culture box (Heal Force Bio-Meditech, Shanghai, China). Cells were collected when reaching 80% overlap.

### 4.2. SEP Extraction and ACN Precipitation

A lysis buffer containing 50 mM HCl, 0.5% dithiothreitol (DTT), and 0.1% N-Dodecyl-β-D-maltoside (DDM) was used for the extraction of SEPs from 4 × 10^7^ total cells. Cell lysates were sonicated for 5 min on ice with an output of 130 W (Biosafer 1200-98C, Nanjing Safer Biotech Co., Ltd., Nanjing, China). The whole-cell lysate was centrifuged (12,000 g at 4 °C for 15 min) to remove cell debris and precipitated proteins caused by acid denaturation. Half of the cell lysate was further incubated with 3.2 vol chilled acetonitrile to deposit high molecular weight protein. All these lysate solutions were further processed with chloroform extraction to remove lipids and some other biological matrices. All the clear supernatant was transferred to a new tube and then evaporated to dryness at low temperature in a SpeedVac.

### 4.3. Offline Fractionation of SEPs by SPE and High pH Reversed-Phase Separation

Self-packed C18 columns were prepared by packing 100 mg of C18 and C8 materials (40–75 μm, 100-Å pore size, Agela Technologies, Beijing, China) into 1mL SPE columns, respectively. The columns were washed 3 times with 1 mL of 100% ACN and then equilibrated with the same volume of 0.1% formic acid (FA). After the peptides had been sequentially loaded onto 3 self-packed C18 columns, the columns were washed 3 times with 1 mL of 0.1% FA for sample desalting and eluted with a series of elution buffers (0.1 mL) containing 0.1% FA with different concentrations of ACN (5%, 10%, 15%, 20%, 25%, 30%, 40%, and 75%). We adopted the high pH reversed-phase separation by StageTip micro-columns from a published protocol with a small modification [37]. Briefly, micro-columns containing 8 disks of Empore C18 material were constructed, activated, and equilibrated as previously reported. After sample loading, the flow-through was collected and desalted. We prepared 9 elution buffers with 5%, 10%, 15%, 20%, 25%, 30%, 40%, 50%, and 75% ACN in 25 mM NH_4_FA. In total, 10 fractions were collected, and the last 4 fractions contained some larger peptides with a mass range from 6 to 12 kDa. All these eluted solutions were freeze-dried and resuspended for the next step, LC/MS/MS analysis.

### 4.4. LC-MS/MS Analysis

Each fraction was analyzed by LC/MS/MS using an Easy-nLC1200 and a Q Exactive plus mass spectrometer (Thermo Scientific, Bremen, Germany). Peptide separation was performed with linear gradients at the flow rate of 300 nL/min (A: 5% ACN/0.1% FA in water, B: 90% ACN/0.1% FA) as follows: 5 min of 5% buffer B, 95 min gradient from 5–40% buffer B, 15 min gradient from 40% to 100% min buffer B to wash the column, 5 min gradient from 100% to 0% buffer B and a final 5 min of 100% buffer A to equilibrate the column. The mass spectrometer was operated in positive ion mode with data-dependent acquisition. Full precursor scans were collected with m/z range from 350 to 2000 at 70 k resolution.

The 20 most abundant ions per scan were selected for MS/MS with an isolation window of 1.8 m/z, normalized higher-energy collisional dissociation (HCD) energy of 27, and resolution of 17.5 k. Maximal fill times were 50 ms for MS or MS/MS scans. Dynamic exclusion was enabled for 40 s and unassigned singly charged ions. Automatic gain control (AGC) was 1 × 10^6^ and 5 × 10^5^. All other parameters remained unchanged. To improve higher mass peptide identification, we performed the second MS/MS acquisition with the following parameter modifications: charge unassigned +1 and +2, AGC increased to 3 × 10^6^, and maximal fill times increased to 200 ms.

### 4.5. Synthesized Peptide LC/MS/MS

In order to verify the accuracy of the identified peptides of SEPs, peptides VDQEIVNIIQER, LLLPCLLSSR, NDTLLSLLK, GSQTLGPR, VNLFNVPK, NVTQHAVGIVLN, and MLALKR were synthesized by Bankpeptide Bio-Tech-Co., Ltd. (Hefei, China). Peptide mixtures containing these synthesized peptides were diluted into 0.01 μg/μL and then analyzed by the same method as described above.

### 4.6. Data Processing

An integrated protein database was generated by adding the homo canonical proteins (UniProt, 20200810) and a collection of protein products from the novel predicted sORF (OpenProt database 1.5 version, 20200625). MS files were processed against the database using Proteome Discover (2.1 version, Thermo Scientific, San Jose, CA, USA) with the following parameters: enzyme: trypsin (full); precursor mass tolerance: 10 ppm; fragment mass tolerance: 0.02 Da; dynamic modification: oxidation (M)/ acetyl (protein N-terminus). The percolator node was used for false discovery rate (FDR) calculation, and a target protein FDR of 0.01 was sought. We also utilized the pFind 3 Open search engine (http://pfind.ict.ac.cn/software/pFind/) to identify microprotein-derived peptides by an open search strategy, which allowed for many variable modifications using the same database and false discovery rate. Some proteins with mass approximates at or above 10 kDa were found in the fraction with high ACN proportion. MS data generated from the fractions containing ACN above 40% were further processed with TopPIC (1.3 version, https://www.toppic.org/software/toppic/index.html).

All the characteristics of the identified SEPs, including MS evidence, translation evidence Kozak motif, domain, and conservation, were in accordance with the OpenProt database [4].

De novo peptide sequencing of the previous bottom-up data was conducted using pNovo (3.1 version, http://pfind.ict.ac.cn/software/pNovo/index.html) with the following parameters: enzymes set as Trypsin KR_C; precursor and fragment tolerances were 10 ppm and 0.02 Da; “carbamidomethyl[C]” as fixed modification and acetyl[AnyN-term] as variable modifications, and the “Open Search” as “false”. We set the “Top-1 Result” to obtain the peptide sequence with the highest score compared to other candidate peptides. Based on the precision-recall analysis, peptides with scores of less than 70 were deleted. Furthermore, the remaining peptides with an identical sequence to the known proteins in UniProt were also deleted.

The selected de novo peptides were mapped back to the genome sequence with ACTG tool [38]. The input file for genome mapping included a FASTA file of the peptides to be searched and a FASTA file of the homo genome. The genome translated in all 6 reading frames, further confirming these candidate peptides by using an embedded peptide validation strategy.

## Data Availability

MS raw data will be available online with ID IPX0003030001 (https://www.iprox.org/).

## Supplementary Materials

The Supplementary Materials are available online at https://www.mdpi.com/article/10.3390/ijms22115476/s1.

## References

[1] Andrews S.J., Rothnagel J.A. (2014). Emerging evidence for functional peptides encoded by short open reading frames. Nat. Rev. Genet..

[2] Plaza S., Menschaert G., Payre F. (2017). In Search of Lost Small Peptides. Annu. Rev. Cell Dev. Biol..

[3] Aebersold R., Agar J.N., Amster I.J., Baker M.S., Bertozzi C.R., Boja E.S., Costello C., Cravatt B.F., Fenselau C., Garcia B.A. (2018). How many human proteoforms are there?. Nat. Chem. Biol..

[4] Brunet M.A., Lucier J.-F., Levesque M., Leblanc S., Jacques J.-F., Al-Saedi H.R.H., Guilloy N., Grenier F., Avino M., Fournier I. (2021). OpenProt 2021: Deeper functional annotation of the coding potential of eukaryotic genomes. Nucleic Acids Res..

[5] Hao Y., Zhang L., Niu Y., Cai T., Luo J., He S., Zhang B., Zhang D., Qin Y., Yang F. (2017). SmProt: A database of small proteins encoded by annotated coding and non-coding RNA loci. Brief. Bioinform..

[6] Olexiouk V., Crappé J., Verbruggen S., Verhegen K., Martens L., Menschaert G. (2016). sORFs.org: A repository of small ORFs identified by ribosome profiling. Nucleic Acids Res..

[7] Pauli A., Norris M.L., Valen E., Chew G.L., Gagnon J.A., Zimmerman S., Mitchell A., Ma J., Dubrulle J., Reyon D. (2014). Tod-dler: An embryonic signal that promotes cell movement via Apelin receptors. Science.

[8] Anderson D.M., Anderson K.M., Chang C.L., Makarewich C.A., Nelson B.R., McAnally J.R., Kasaragod P., Shelton J.M., Liou J., Bassel-Duby R. (2015). A micropeptide encoded by a putative long non-coding RNA regulates muscle per-formance. Cell.

[9] Huang J.-Z., Chen M., Chen D., Gao X.-C., Zhu S., Huang H., Hu M., Zhu H., Yan G.-R. (2017). A Peptide Encoded by a Putative lncRNA HOXB-AS3 Suppresses Colon Cancer Growth. Mol. Cell.

[10] Stein C.S., Jadiya P., Zhang X., McLendon J.M., Abouassaly G.M., Witmer N.H., Anderson E.J., Elrod J.W., Boudreau R.L. (2018). Mitoregulin: A lncRNA-Encoded Microprotein that Supports Mitochondrial Supercomplexes and Respiratory Efficiency. Cell Rep..

[11] Bazzini A.A., Johnstone T.G., Christiano R., Mackowiak S.D., Obermayer B., Fleming E.S., Vejnar C.E., Lee M.T., Rajew-sky N., Walther T.C. (2014). Identification of small ORFs in vertebrates using ribosome footprinting and evolu-tionary conservation. EMBO J..

[12] Ingolia N.T., Brar G.A., Stern-Ginossar N., Harris M.S., Talhouarne G.J., Jackson S.E., Wills M.R., Weissman J.S. (2014). Ribo-some profiling reveals pervasive translation outside of annotated protein-coding genes. Cell Rep..

[13] Slavoff S., Mitchell A.J., Schwaid A.G., Cabili M.N., Ma J., Levin J.Z., Karger A.D., Budnik B.A., Rinn J.L., Saghatelian A. (2012). Peptidomic discovery of short open reading frame–encoded peptides in human cells. Nat. Chem. Biol..

[14] Samandi S., Roy A.V., Delcourt V., Lucier J.-F., Gagnon J., Beaudoin M.C., Vanderperre B., Breton M.-A., Motard J., Jacques J.-F. (2017). Deep transcriptome annotation enables the discovery and functional characterization of cryptic small proteins. eLife.

[15] Chen J., Brunner A.-D., Cogan J.Z., Nuñez J.K., Fields A.P., Adamson B., Itzhak D.N., Li J.Y., Mann M., Leonetti M.D. (2020). Pervasive functional translation of noncanonical human open reading frames. Science.

[16] Ma J., Diedrich J.K., Jungreis I., Donaldson C., Vaughan J., Kellis M., Yates J.R., Saghatelian A. (2016). Improved Identification and Analysis of Small Open Reading Frame Encoded Polypeptides. Anal. Chem..

[17] D’Lima N.G., Ma J., Winkler L., Chu Q., Loh K.H., Corpuz E.O., Budnik B.A., Lykke-Andersen J., Saghatelian A., Slavoff S.A. (2017). A human microprotein that interacts with the mRNA decapping complex. Nat. Chem. Biol..

[18] Yin X., Jing Y., Xu H. (2019). Mining for missed sORF-encoded peptides. Expert Rev. Proteom..

[19] Ma J., Ward C.C., Jungreis I., Slavoff S.A., Schwaid A.G., Neveu J., Budnik B.A., Kellis M., Saghatelian A. (2014). Discovery of human sORF-encoded polypeptides (SEPs) in cell lines and tissue. J. Proteome Res..

[20] Toby T.K., Fornelli L., Kelleher N.L. (2016). Progress in Top-Down Proteomics and the Analysis of Proteoforms. Annu. Rev. Anal. Chem..

[21] Blank-Landeshammer B., Teichert I., Märker R., Nowrousian M., Kück U., Sickmann A. (2019). Combination of Proteogenomics with PeptideDe NovoSequencing Identifies New Genes and Hidden Posttranscriptional Modifications. mBio.

[22] Wang B., Hao J., Pan N., Wang Z., Chen Y., Wan C. (2021). Identification and analysis of small proteins and short open reading frame encoded peptides in Hep3B cell. J. Proteom..

[23] Baghdady Y.Z., Schug K.A. (2019). Online Comprehensive High pH Reversed Phase × Low pH Reversed Phase Approach for Two-Dimensional Separations of Intact Proteins in Top-Down Proteomics. Anal. Chem..

[24] Dahl L.D., Corydon T.J., Rankel L., Nielsen K.M., Füchtbauer E.-M., Knudsen C.R. (2014). An eEF1A1 truncation encoded by PTI-1 exerts its oncogenic effect inside the nucleus. Cancer Cell Int..

[25] Bogaert A., Fernandez E., Gevaert K. (2020). N-Terminal Proteoforms in Human Disease. Trends Biochem. Sci..

[26] Secher A., Kelstrup C.D., Conde-Frieboes K.W., Pyke C., Raun K., Wulff B.S., Olsen J.V. (2016). Analytic framework for pep-tidomics applied to large-scale neuropeptide identification. Nat. Commun..

[27] Sanders W.S., Wang N., Bridges S.M., Malone B.M., Dandass Y.S., McCarthy F.M., Nanduri B., Lawrence M.L., Burgess S.C. (2011). The Proteogenomic Mapping Tool. BMC Bioinform..

[28] Laumont C.M., Daouda T., Laverdure J.-P., Bonneil É., Caron-Lizotte O., Hardy M.-P., Granados D.P., Durette C., Lemieux S., Thibault P. (2016). Global proteogenomic analysis of human MHC class I-associated peptides derived from non-canonical reading frames. Nat. Commun..

[29] Chew G.-L., Pauli A., Schier A.F. (2016). Conservation of uORF repressiveness and sequence features in mouse, human and zebrafish. Nat. Commun..

[30] Ruiz-Orera J., Albà M.M. (2019). Translation of Small Open Reading Frames: Roles in Regulation and Evolutionary Innovation. Trends Genet..

[31] Hellens R.P., Brown C., Chisnall M.A., Waterhouse P.M., Macknight R.C. (2016). The Emerging World of Small ORFs. Trends Plant Sci..

[32] Dumesic P.A., Egan D.F., Gut P., Tran M.T., Parisi A., Chatterjee N., Jedrychowski M., Paschini M., Kazak L., Wilensky S.E. (2019). An Evolutionarily Conserved uORF Regulates PGC1α and Oxidative Metabolism in Mice, Flies, and Bluefin Tuna. Cell Metab..

[33] Ree R., Varland S., Arnesen T. (2018). Spotlight on protein N-terminal acetylation. Exp. Mol. Med..

[34] Macias L.A., Santos I.C., Brodbelt J.S. (2020). Ion Activation Methods for Peptides and Proteins. Anal. Chem..

[35] Muth T., Renard B.Y. (2018). Evaluating de novo sequencing in proteomics: Already an accurate alternative to database-driven peptide identification?. Brief. Bioinform..

[36] Couso J.P., Patraquim P. (2017). Classification and function of small open reading frames. Nat. Rev. Mol. Cell Biol..

[37] Ruprecht B., Zecha J., Zolg D.P., Kuster B. (2017). High pH Reversed-Phase Micro-Columns for Simple, Sensitive, and Efficient Frac-tionation of Proteome and (TMT labeled) Phosphoproteome Digests. Methods Mol. Biol..

[38] Seunghyuk C., Hyunwoo K., Eunok P. (2017). ACTG: Novel peptide mapping onto gene models. Bioinformatics.

