# Supplementary material for: Improved Identification of Small Open Reading Frames Encoded Peptides by Top-Down Proteomic Approaches and De Novo Sequencing
Source: Int J Mol Sci. 2021 May 22;22(11):5476. doi: 10.3390/ijms22115476 (PMC8197016; doi:10.3390/ijms22115476)
Supplement: Supplementary file 1 [file ijms-22-05476-s001.zip › SI_Wang_IJMS.pdf]

Improved Identification of Small Open Reading Frames Encoded Peptides by Top-Down Proteomics Approaches and de Novo Sequencing

Bing Wang, Zhiwei Wang[#], Ni Pan[#], Jiangmei Huang, Cuihong Wan*

Hubei Key Lab of Genetic Regulation and Integrative Biology, School of Life Sciences, Central China Normal University, No. 152 Luoyu Road, Wuhan 430079, P. R. China

Corresponding author:

Prof. Cuihong Wan

Address: No. 152 Luoyu Road, Wuhan, P. R. China, 430079

Phone number: 86-027-67861936

Email: ch_wan@mail.ccnu.edu.cn

# Authors contributed equally to this work.

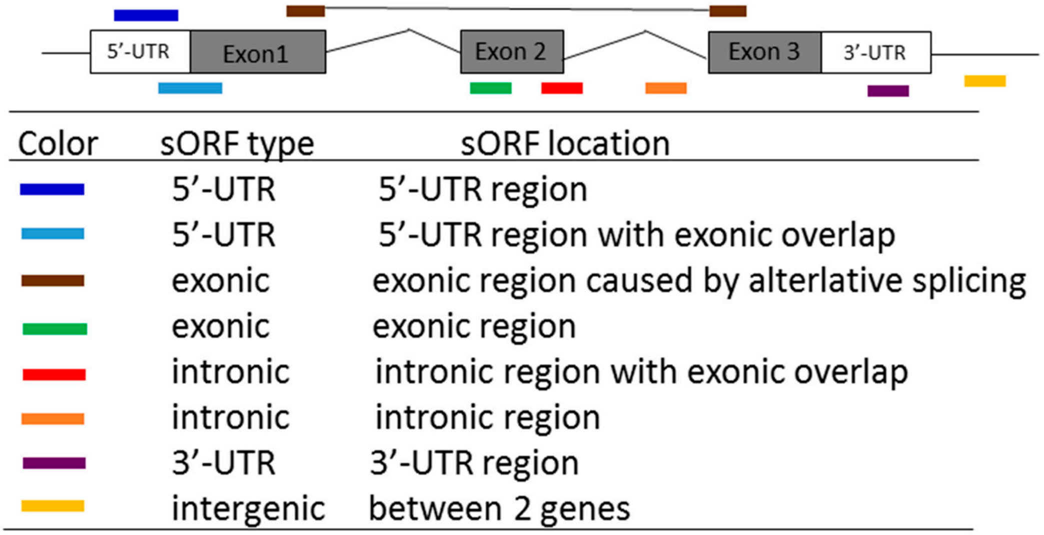

Figure S1. Different sORF types and corresponding genomic location.

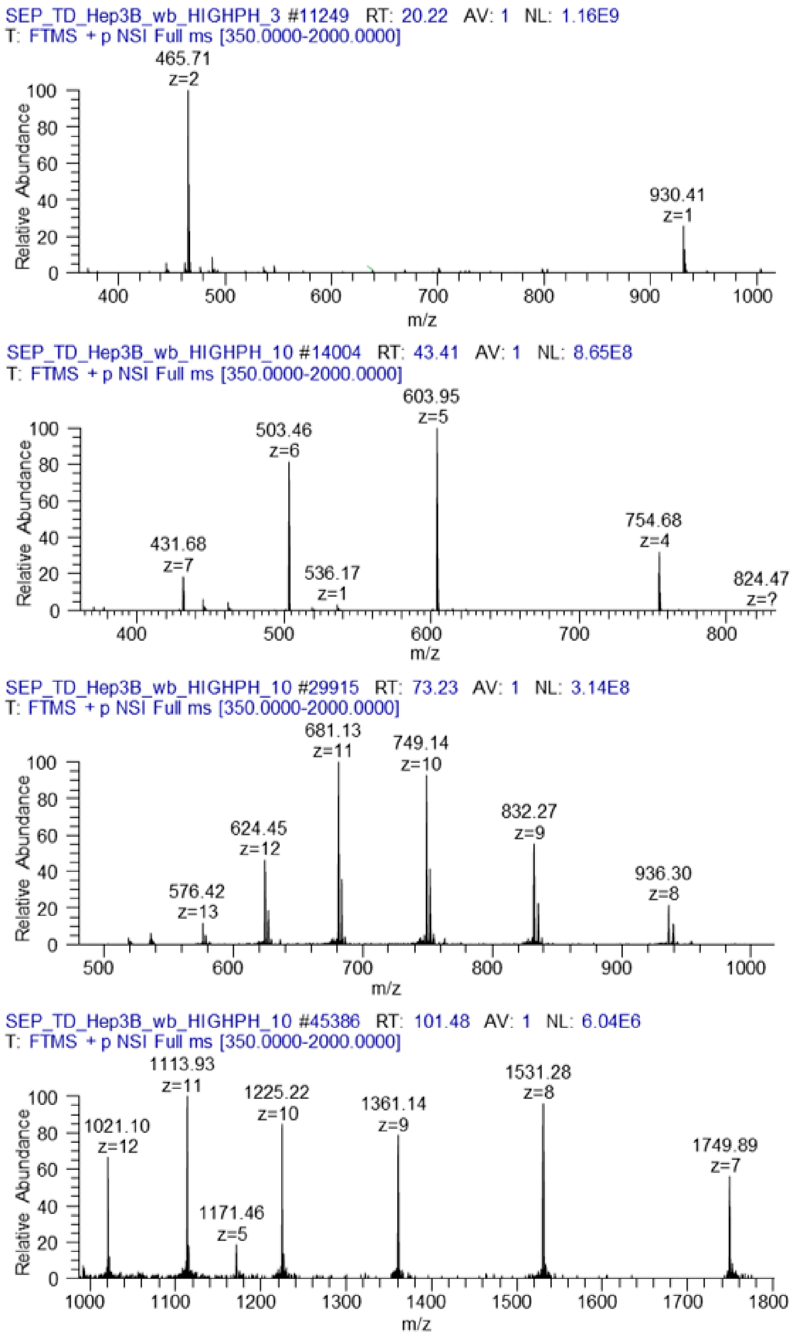

Figure S2. MS1 spectra of extracted peptides.

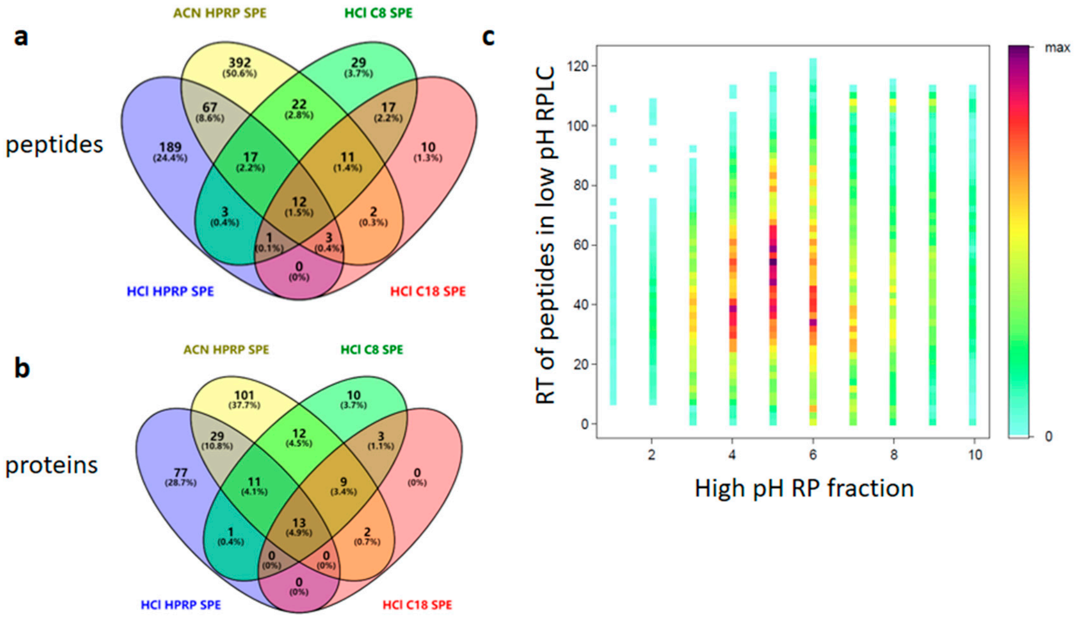

Figure S3. Separation by high pH and low pH reversed-phase chromatography promotes SEPs identification. Venn diagram of (**a**) peptides and (**b**) SEPs number from high pH and low pH reversed-phase chromatography; (**c**) Heat map of peptides intensity across different fractions.

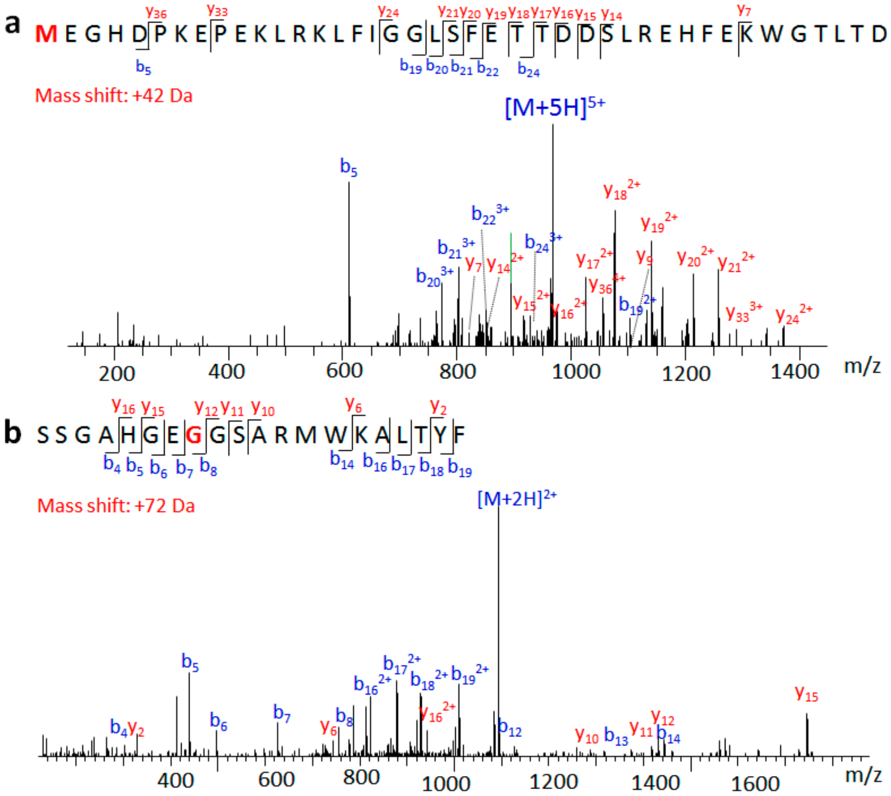

Figure S4. Mass shift of SEPs. (**a**) IP_669887 peptide; (**b**) IP_671398 peptide. The amino acid in red is where the mass shifts occur.

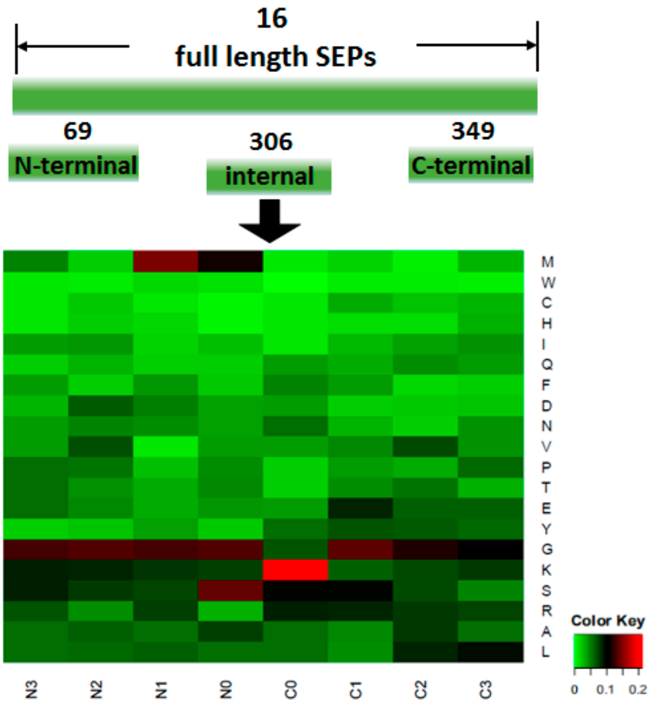

Figure S5. Amino acids in proteolysis cleavage site.

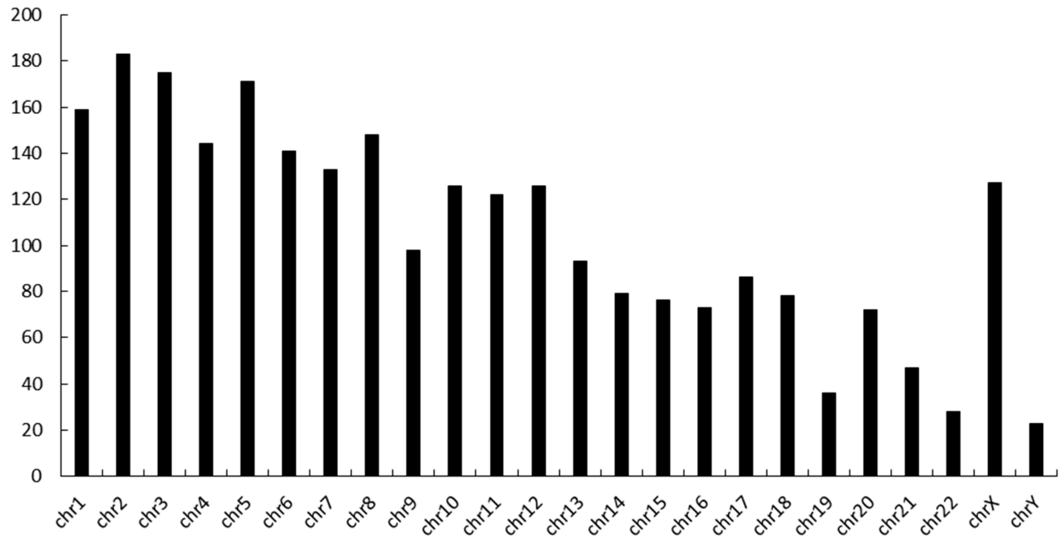

Figure S6. Number of SEPs from de novo sequencing located on each chromosome.

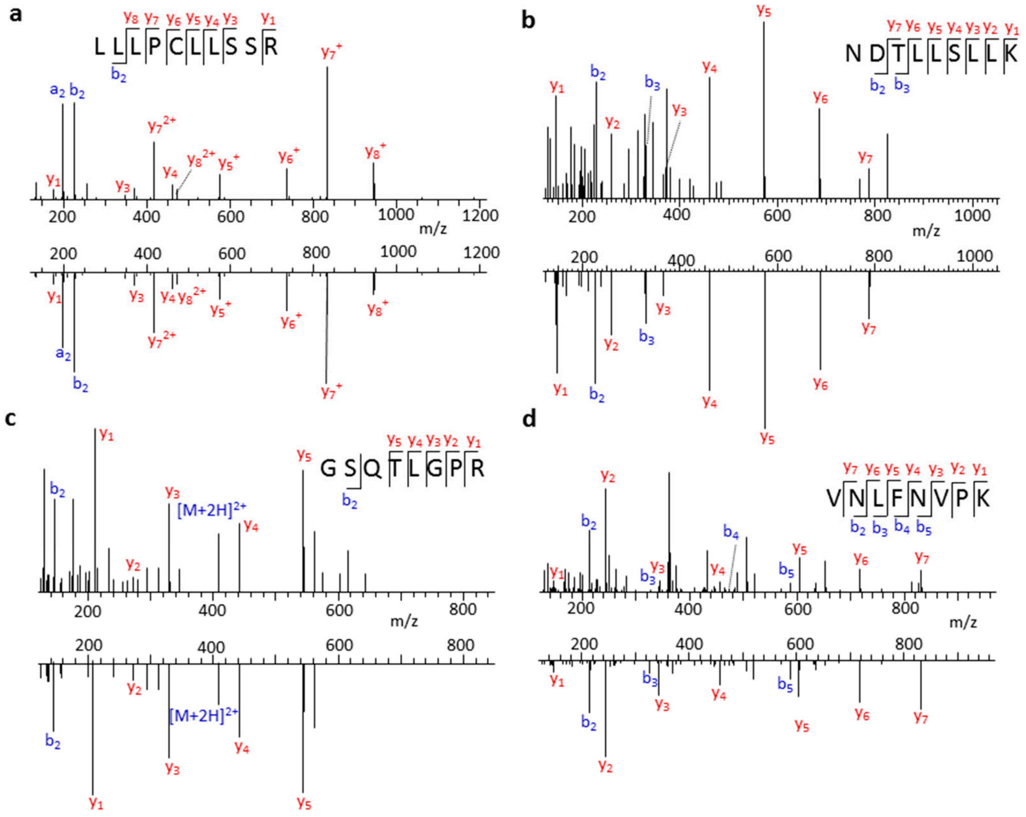

Figure S7. Comparison of the mass spectra of de novo sequencing peptides (up) and their synthetic peptides (below).

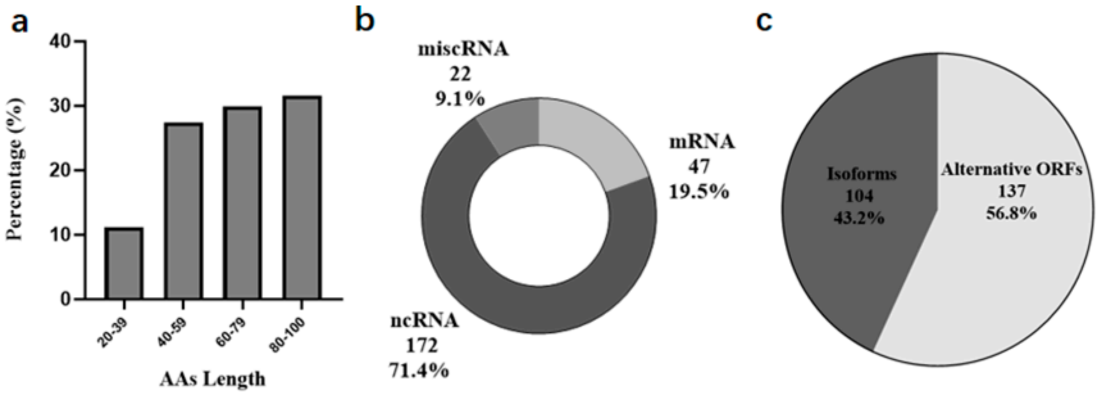

Figure S8. Length distribution (**a**), RNA types (**b**), and ORF types (**c**)of SEPs.

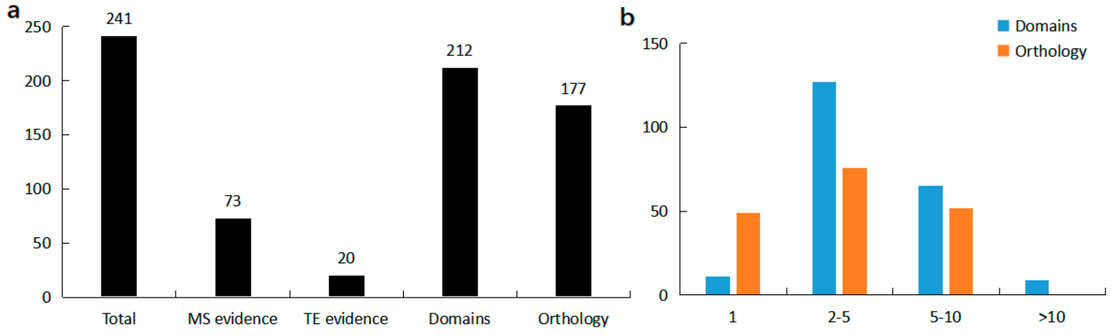

Figure S9. Characteristic analysis of the identified SEPs. (**a**) Number of SEPs that have experiment evidence, domains and orthologous. (**b**) Number distribution of functional domains and conservation species.
